# Implications of President Trump’s Second Term Executive Orders on Global and Public Health

**DOI:** 10.3389/ijph.2025.1608402

**Published:** 2025-02-24

**Authors:** Keita Wagatsuma

**Affiliations:** ^1^ Division of International Health (Public Health), Graduate School of Medical and Dental Sciences, Niigata University, Niigata, Japan; ^2^ Institute for Research Administration, Niigata University, Niigata, Japan

**Keywords:** pandemic preparedness, public health policy, climate change, withdrawal, global health security

## Introduction

On 20 January 2025, Donald J. Trump assumed office for his second term as President of the United States (US), initiating an immediate shift in public health policies [1]. The newly issued executive orders encompass pandemic preparedness, environmental health, and global collaborations, raising concerns regarding their long-term impact. The US government’s retreat from international health partnerships, combined with substantial budget reductions, poses significant threats to global health security.

## Pandemic Preparedness and Research Disruptions

Among the most striking policy reversals is the systematic defunding of key federal agencies tasked with pandemic response. The Department of Health and Human Services (HHS) has imposed stringent restrictions, including a suspension of new contracts, grants, external communications, and medical research reviews, impacting over 13 health agencies [2, 3]. These measures have effectively frozen a $50 billion industry, limiting travel for public health officials to life-threatening situations only, thereby impeding outbreak responses—particularly concerning the ongoing highly pathogenic H5N1 avian influenza (HPAI H5N1) viruses’ crisis [3]. The National Institutes of Health (NIH) has also ceased new funding for research initiatives, disrupting coronavirus disease 2019 (COVID-19) research and broader scientific endeavors. Furthermore, halting financial support for global projects threatens international collaborations on infectious disease surveillance. Indeed, without advisory committee meetings, the NIH is unable to issue research grants, effectively freezing 80% of its $47 billion budget, which supports research initiatives both domestically and internationally. Key agencies such as the Centers for Disease Control and Prevention (CDC) and the Food and Drug Administration (FDA) also face these new mandates amidst leadership instability, with key positions remaining unfilled.

The implications of these measures extend beyond US borders. President Trump has also signed an executive order to withdraw the United States from the World Health Organization (WHO), citing concerns over national sovereignty [4]. This withdrawal threatens to destabilize global health initiatives, particularly in Africa, where many countries rely on US funding through WHO-led programmers. The WHO has warned that this departure will undermine disease surveillance, polio eradication efforts, and emergency outbreak responses, particularly in low-income regions. With the US historically contributing 15% of WHO’s total budget, the financial gap left by its departure—approximately $1.28 billion annually—jeopardizes the health security of millions worldwide [5].

## Climate Change, Environmental Rollbacks, and Health Implications

President Trump’s executive orders have also reversed environmental policies designed to mitigate climate change, including the withdrawal from the Paris Agreement and the deregulation of fossil fuel industries. Recent estimates suggest that climate-related health risks—such as vector-borne diseases (e.g., dengue), respiratory illnesses, and extreme weather mortality—could contribute to an additional 250,000 deaths annually by 2050 [6]. By dismantling emissions restrictions on coal-fired power plants, these policy reversals are expected to increase air pollution-related deaths by tens of thousands per year in the US alone [7, 8]. Furthermore, the dismantling of environmental health policies disproportionately affects marginalized populations, including low-income urban communities, agricultural workers, and indigenous groups. These populations bear the brunt of climate-exacerbated health conditions, including heat stress, food insecurity, and increased exposure to airborne pollutants.

## Erosion of Global Health Collaboration

Trump’s “America First” approach extends to a sharp decline in international health funding. In tandem with the WHO withdrawal, the Trump administration has enacted a comprehensive freeze on new funding for most foreign aid programs worldwide, excluding emergency food aid and military assistance to Israel and Egypt [9]. This suspension affects billions of dollars allocated for health, education, development, and other aid initiatives, with critical health programs like the President’s Emergency Plan for acquired immunodeficiency syndrome (AIDS) Relief (PEPFAR) facing significant uncertainties. The halt in funding jeopardizes ongoing efforts to combat human immunodeficiency virus (HIV)/AIDS, tuberculosis, malaria, and other infectious diseases. Furthermore, the reinstatement of the Mexico City Policy, which prevents international non-governmental organizations that perform or promote abortions from receiving federal funding, has broader implications for global health collaboration [10]. This policy not only restricts reproductive health services but also disrupts integrated health programs that address a range of issues, including maternal and child health and gender-based violence. The policy’s reinstatement may lead to service gaps and strained relationships between the US and international health partners.

### Conclusion

The executive orders issued in the early days of President Trump’s second term indicate a significant shift in US public health policy, with potential long-term consequences for both domestic and global health. The withdrawal from the WHO could disrupt international disease surveillance and outbreak response efforts, while budget cuts to domestic health agencies risk undermining pandemic preparedness and research initiatives. Furthermore, climate policy reversals may disproportionately impact vulnerable populations, exacerbating existing health inequalities. As these policies unfold, continuous assessment and international cooperation will be crucial in mitigating their impact. Global health institutions and policymakers must mobilize alternative funding strategies, reinforce multilateral collaborations, and advocate for science-driven public health policies. The erosion of global health governance under the Trump administration is not merely a domestic concern but a global emergency demanding an urgent and coordinated response. Global health stakeholders must engage in strategic discussions to ensure that public health infrastructure remains resilient in the face of these changes.
